# Data on the effects of cellulase hydrolysis, acid treatment and particle size distribution on physicochemical and functional properties of coconut (cocos nucifera L) cake dietary fibres

**DOI:** 10.1016/j.dib.2018.08.018

**Published:** 2018-08-11

**Authors:** Yajun Zheng, Yan Li

**Affiliations:** a College of Food Science, Shanxi Normal University, Linfen 041004, China; b College of Life Sciences and Food Engineering of Hebei Engineering University, Handan 056038, China

**Keywords:** Defatted coconut dietary fiber, Cellulase, Acidic treatment, Monosaccharide composition, Surface area

## Abstract

The data presented in this article are related to the research article entitled “Physicochemical and functional properties of coconut (Cocos nucifera L) cake dietary fibres: Effects of cellulase hydrolysis, acid treatment and particle size distribution” [1]. This article describes the effect of acidic treatment, cellulase hydrolysis and particle size distribution on the monosaccharide composition, X-ray diffraction, Fourier-transformed infrared and spectroscopy surface area of coconut cake dietary fiber. The field data set is made publicly available to the potential re-use of coconut cake or other plants by-products.

**Specifications Table**

**Table:** 

Subject area	Physics, Chemistry
More specific subject area	Physicochemical properties of dietary fibers
Type of data	Table, image (x-ray), text file, graph, figure
How data was acquired	Survey (a NS800 spectrocolorimeter, Shenzhen 3NH TECHNOLGOY CO. LTD., China; a Laser Diffraction Particle Size Analyzer, MS3000, Malvern instruments Ltd., UK),
	SEM (S-3400 scanning electron microscope, Hitachi, Ltd., Tokyo, Japan),
	X-ray diffractometer (D8, Brucker AXS GMBH, Germany),
	Fourier-transformed infrared spectroscopy (Tensor 27 spectrometer, Bruker, Germany).
Data format	Raw, filtered
Experimental factors	DCC, defatted coconut cake; DCCDF, dietary fiber produced from defatted coconut cake with α-amylase, papain and glucoamylase; DCCDF-A, defatted coconut cake dietary fiber treated by acid; DCCDF-C, defatted coconut cake dietary fiber with cellulase hydrolysis
Experimental features	Measured the relationship between cellulase hydrolysis, acidic treatment and particle size and the physicochemical properties of defatted coconut cake dietary fiber.
Data source location	China
Data accessibility	The data are available with this article
Related research article	Physicochemical and functional properties of coconut (Cocos nucifera L) cake dietary fibres: Effects of cellulase hydrolysis, acid treatment and particle size distribution. Food Chemistry.2018 257: 135–142 [1].

**Value of the data**

• The data provide the potential re-use of coconut cake or other plants by-products.
• The data provide information on how to improve some functional properties of defatted coconut cake dietary fiber.
• This data allows other researchers to extend the statistical analyses.

## 1 Data

**Fig. 1:**
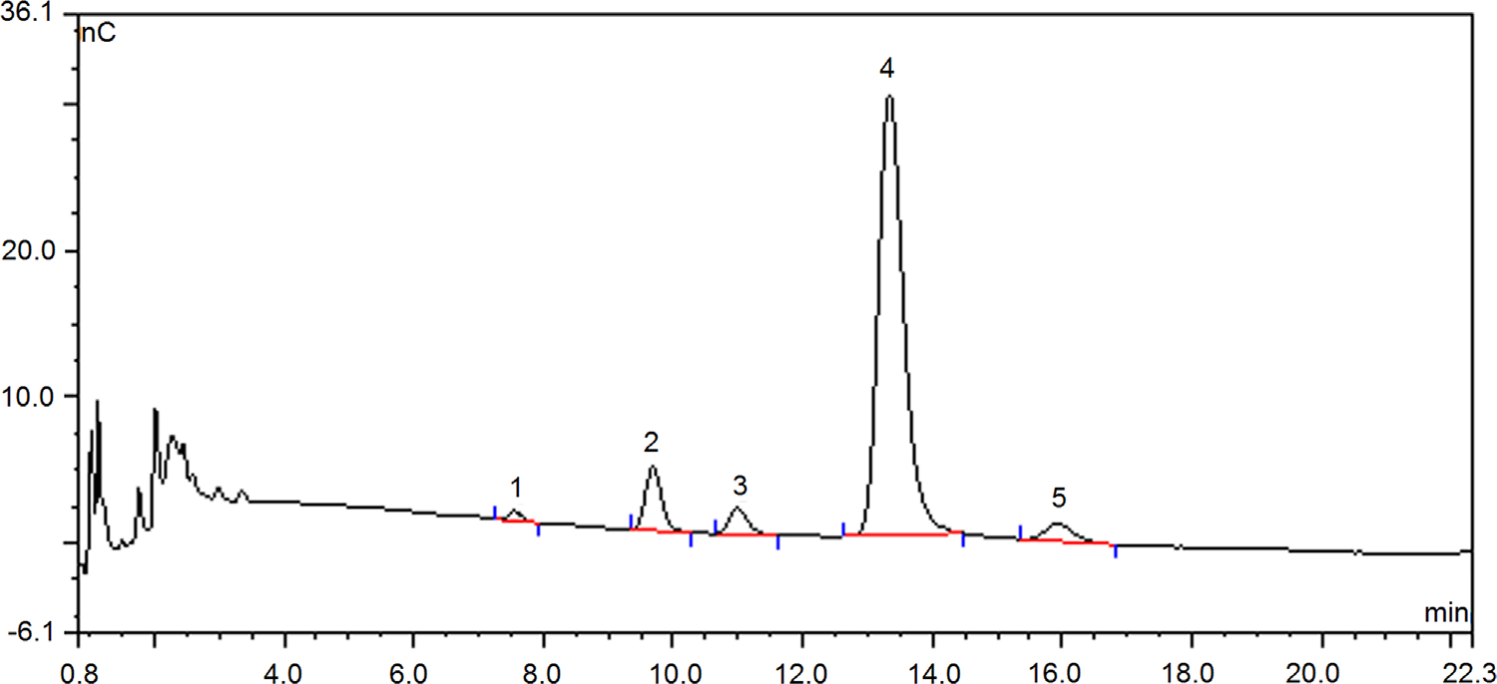
Chromatography profile on monosaccharide composition of defatted coconut cake by ion chromatography Peak 1- L-Arabinose, 2- D-galactose, 3- glucose, 4- xylose, 5- fructose.

**Fig. 2:**
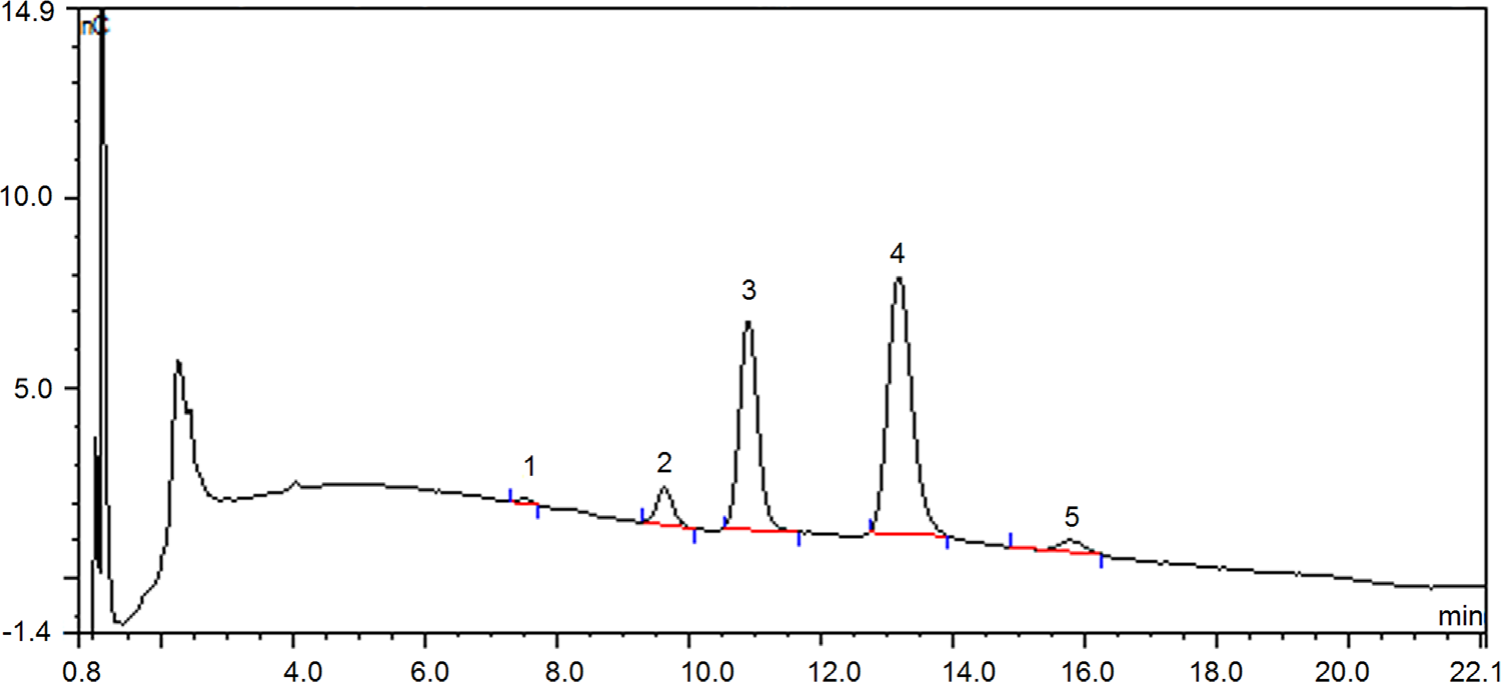
Chromatography profile on monosaccharide composition of defatted coconut cake dietary fiber (DCCDF) by ion chromatography Peak 1- L-Arabinose, 2- D-galactose, 3- glucose, 4- xylose, 5- fructose.

**Fig. 3:**
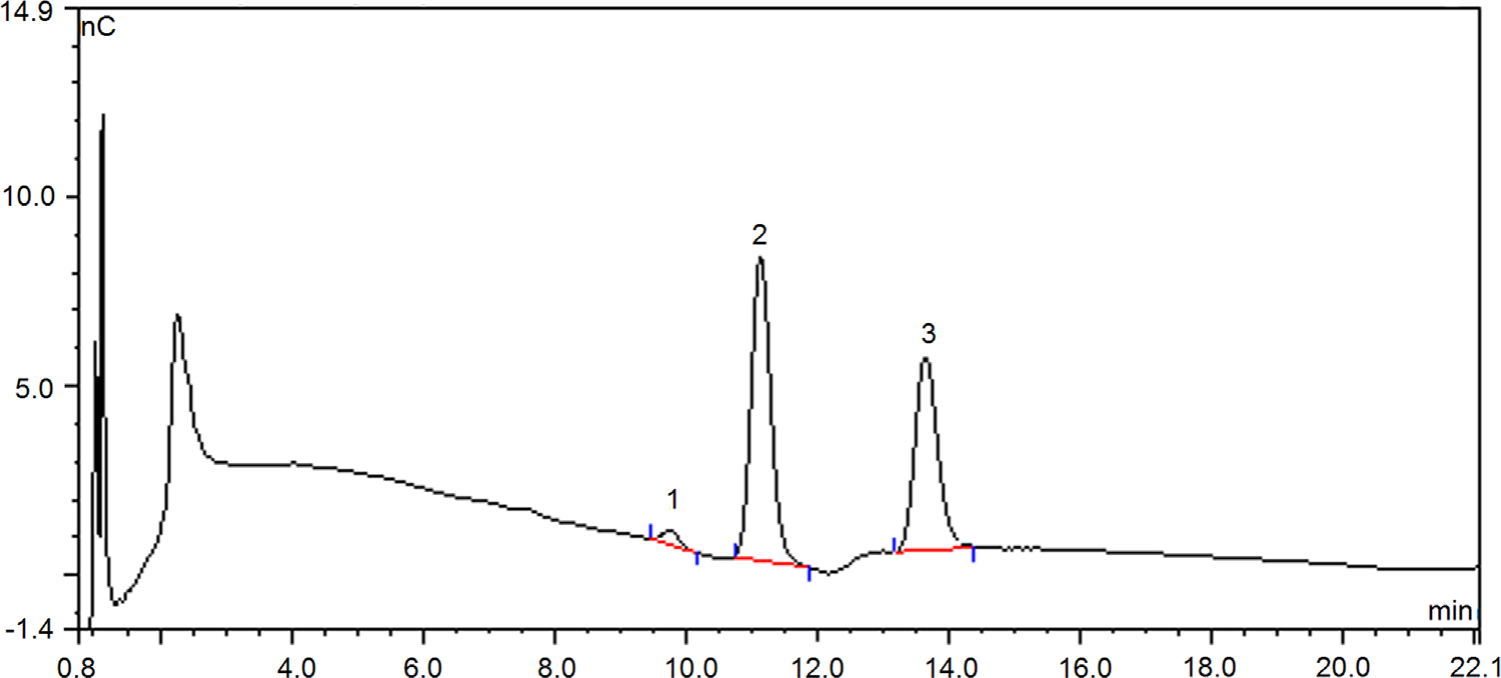
Chromatography profile on monosaccharide composition of defatted coconut cake dietary fiber treated by acid (DCCDF-A) by ion chromatography Peak 1- D-galactose, 2- glucose, 3- xylose.

**Fig. 4:**
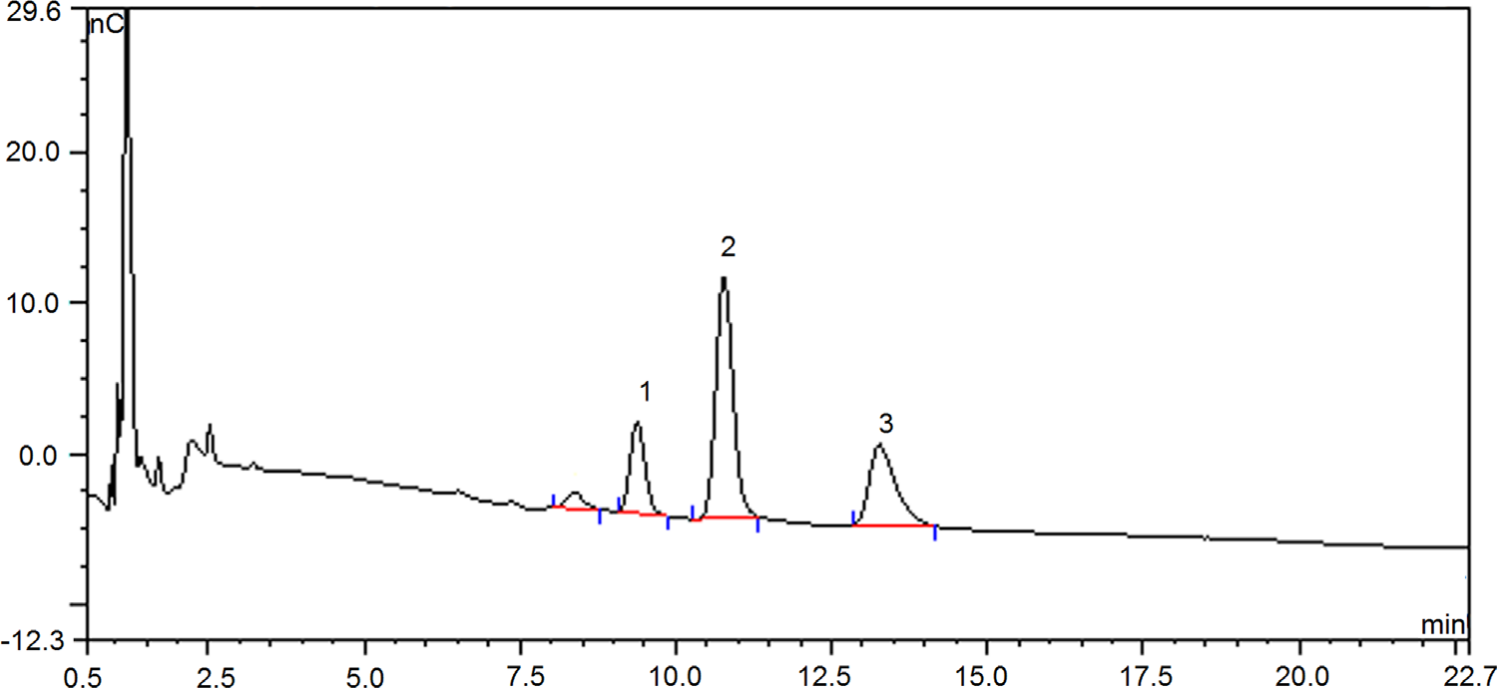
Chromatography profile on monosaccharide composition of defatted coconut cake dietary fiber treated by cellulase (DCCDF-C) by ion chromatography Peak 1- D-galactose, 2- glucose, 3- xylose.

The Fig. 1, Fig. 2, Fig. 3, Fig. 4 show the monosaccharide composition of defatted coconut cake dietary fibers. The X-ray diffraction and Fourier-transformed infrared spectroscopy can be seen in the Ref [1] and Ref [2], respectively. Moreover, relationship between particle size and surface area was shown in Ref [3].

## 2 Experimental design, materials and methods

The experiments was carried out to determine the relationship between cellulase hydrolysis, acid treatment and particle size distribution and the monosaccharide composition, X-ray diffraction, Fourier-transformed infrared and spectroscopy surface area of coconut cake dietary fiber. Firstly, defatted coconut cake dietary fiber (DCCDF) was prepared from defatted coconut cake with α-amylase, papain and glucoamylase. Then DCCDF was subjected to acidic treatment and cellulase hydrolysis respectively, and defatted coconut cake dietary fiber treated by acid (DCCDF-A) and defatted coconut cake dietary fiber treated by cellulase hydrolysis (DCCDF-C) were obtained. Secondly, the chemical composition, particle size distribution, color, surface and microstructure, X-ray diffraction and Fourier-transformed infrared spectroscopy of DCCDF, DCCDF-A, DCCDF-C were determined [2], [3], [4], [5], [6].
