# Supplementary material for: Data on the effects of cellulase hydrolysis, acid treatment and particle size distribution on physicochemical and functional properties of coconut (cocos nucifera L) cake dietary fibres
Source: Data Brief. 2018 Aug 11;20:521–4. doi: 10.1016/j.dib.2018.08.018 (PMC6126078; doi:10.1016/j.dib.2018.08.018)
Supplement: Supplementary file 1 — Supplementary material [file mmc1.docx]

**Conflict of Interest Form**

Authors claim no conflict of interest.
